# Rules of Hygiene

**Published:** 1870-08

**Authors:** 


### EDITORIAL DEPARTMENT.

---

*Rules of Hygiene.*—Dr, Geo. H. Napheys, author of "Modern Therapeutics," who is now in England, sends the following "Rules of Hygiene" in a letter to the *Medical and Surgical Reporter*. These rules are disposed along the walls and columns of the Free Public Library and Reading Room, having been compiled by seven prominent medical men connected with the principle hospital in London and Liverpool.

I. Wash the whole body, once at least in every forty-eight hours, either with cold or slightly warmed water, and rub thoroughly dry with a rough towel.

II. Eat your food *slowly* and chew it well, and sit still at least ten minutes after every meal; so may you hope to preserve both your teeth and your digestion. Beware of drinking any *very* hot fluids; tea and coffee should stand till tolerably cool. After fatigue and long fasts hot fluids, only not *too* hot, are valuable, and a few mouthfuls taken in such cases, before beginning to eat are very proper, especially for elderly people.

III. Turn all your clothes (particularly those which you wear next to your skin) inside out before you go to bed, and hang them up to air above the level of your head.

If possible have two suits of clothes, and wear them on alternate days, folding up carefully those not in use after they are aired.

IV. Be sure to secure fresh air in your bedroom during the night.

V. If you have no ventilator in the window, or ceiling, or roof, or over the door, then leave the window from half an inch to two or three inches open during the night. Of course take care that there be no strong draft blowing in upon you during the night; but any thing is better than converting your bedroom into a black hole of Calcutta. Boring four or five tolerably large holes, an inch or so in diameter, through the *bottom* of your bedroom door will go a long way towards keeping the atmosphere sweet during the night, If you can, provide some means of escape for the foul air by a small aperture through the ceiling.

VI. Eschew ,if you care for your teeth, all sweets, tarts, pastry, and confectionary, and also much sugar.

VII. Never sit down to breakfast without first going out into the open air for at least *three* or *four* minutes. Make your walks longer or shorter, according to your health and strength.

VIII. Open your bed entirely, lifting the sheet and blanket on which you have been lying, so as to let the air get underneath ; and leave the window open, top and bottom, when you quit your bedroom in the morning.

IX. Do not eat more than *four* good meals a day ; the chances are that you will find your appetite and digestion benefited by taking only three, and, perhaps even only two, hearty meals a day. Most especially avoid eating and drinking between meals.

X. Do not miss any chance of learning to *ride*, to *swim*, to *fence*, to *play single stick*, to *play cricket*, and *foot-ball*, to *row*, and to *spar*.

XI. Get, if you can, a plunge bath (a cold saltwater bath is best), the first thing in the morning, twice or thrice a week.

XII. Do not plaster down your hair with hog's lard, falsely called "pomatum," "pomade," or "bear's grease ;" the hair is meant to assist in carrying off perspiration, and should not be clogged with grease. No appreciable mischief results from *oiling* the hair, if you like to do so ; nor does sweet oil do any harm to any part of the body, if you like to use it, by rubbing it into the skin before a fire, but on the contrary good, as it renders the limbs supple and more capable of strong muscular exertion.

Animal.oils are better and less drying than vegetable ones, such as olive oil and cocoanut oil, for either skin or hair, and perhaps marrow oil is the best of all. The best sweet salad oil does no harm to any part of the body, but, on the contrary, good.

XIIl. Brush your teeth the last thing every night before going to bed, and comb and brush your hair the wrong way, so as to let the air in upon your head.

Rinse out the mouth after every meal.

XIV. If you have a flesh brush, use it once a day. The best time is at night. If not, polish your skin with a rough towel before you go to bed.

XV. If you dine out, avoid drinking more than one kind of wine; as a rule, after dinner, drink no wine, but take instead, half an hour afterwards, a cnp of good strong black coffee without milk or sugar, except, perhaps, with the smallest bit of this last. Take nothing after this the same night, if it be a late dinner you have had.

It is best neither to take your "café noir" (small cup of black coffee) nor to smoke tobacco till at least half an hour after meals.

XVI. 1f you arc troubled with cold feet at night, use plenty of friction (or rubbing), before getting into bed :and if that does not answer, then sponge them with cold water, and, when drying them, rub the toes and ankles upward, and not downward. In case this plan fails, as it does sometimes, and the feet remain still deadly cold, then try putting them in a mustard foot-bath before stepping into bed, and put on a pair of thick dry woolen socks directly afterward. The socks can be removed as soon as the feet are warm.

XVII. If you are troubled with costiveness or constipation, try taking the best Scotch oatmeal porridge (only let it be long aud thoroughly boiled), every other morning for breakfast. Use salt and milk, according to taste, and take it, either with or without the addition of treacle, as you find it best agrees with you. Eat brown bread .(that is, bread made of whole ground wheat) and butter.

A glass of cold fresh water taken when you first get out of bed, followed by a run or walk before breakfast, has often an excellent effect in removing heartburn, and in moving the bowels gently, particularly when at sea. Costiveness is a common complaint of landsmen during a sea voyage, and a glass of cold water taken then, an hour before breekfast, frequently saves resorting to the ship's medicine chest.

XVIII. Eschew all hot and heavy suppers, unless you wish for an attack of nightmare, and avoid all suppers, as a rule, unless dinner has been taken early. A so-called "severe tea" late at night, is usually unwholesome. Never go to bed, if you can help it, with an entirely "empty stomach." This is often a cause of "insomnia" or sleeplesness, especially in elderly persons. By an "empty stomach" I mean, when a fast of five or six hours has been observed, and the last meal was not a substantial one. A tablespoonful of Hollands, or so, in half a tumbler of cold water, is very excellent for old people at bed-time, and some plain unleavened biscuit may be eaten at the same time. If you are troubled with a so-called "sour stomach," or heartburn at bed-time, or are in the habit of grinding your teeth when asleep, try eating a bit of stewed French apple (sold at the grocers under the mame of "dried Normandy pippins") just before going to bed, or a stewed prune. Do not get in the habit of taking carbonate of soda in water, for heartburn; for its constant use may prove very injurious to the stomach.

XIX. If you have any one ill with fever, or any infectious disease in the house, do not visit him the first thing in the morning, on an empty stomach; but take a mouthful of good coffee or tea and a crust of bread before entering his bed room.

XX. If you are troubled with sleeplessness, rise early and get a good walk, or better still, a good ride, if you can afford to keep a horse, before breakfast; sponge the whole body before going to bed, and rub dry with a Turkish towel; use the dumb bells or any other gymnastic exercise, and jump into bed warm, and banish unpleasant thoughts. Do not smoke strong tobacco the last thing at night and adopt a warm, red flannel shirt, instead of a, cold cotton or linnen one.

XXI. Look sharp after all drains, middens, privies and cesspools, connected with your house, if you wish to keep out infection, or any low fever of the typhoid class. Take care to keep them in good repair and working order, and flush all sewers and drains now and then with oceans of water. In a dry season pour a pailful or two of water, with about a quarter of a pint of carbolic acid (sold at all chemists as a disinfectant) in it, into all your drains, and cesspools, and middens, every other day, at least, to take away any bad smell. If you cannot get carbolic acid, use chloride of lime, Burnett's disinfecting fluid, or something of the same sort. Any way, get rid of foul smells in your house somehow. To purify a room pour a wine-glass or so full of vinegar on a pan of red-hot cinders, and let the vapor from it fill the room; then open windows and door. As a rule fill your washhand basin wfth water *over night*, against next morning and do not neglect at all times to put half a pint, or so, of water into each chamber utensil, or night stool, in a bed-room. You cannot be too cleanly or airy in a bed-room; banish all carpets, and see that no dirty clothes are left under the bed, and no slop left unemptied. Keep all dirty clothes' bags anywhere you like, only *not* in a bed-room.

XXII. *How to wash yourself in the morning.*—Fill your basin over night, and, unless you take a plunge-bath or shower-bath, as soon as you rise, commence by putting your face deep into the basin; open and shut your eyes two or three times, looking at the bottom of the basin. Take your sponge full of water, and sponge neck and back of the head thoroughly.

I advocate washing the whole head with water. Then turn the whole head on one side, in turns, *and fill each ear with cold water*, shake the head, and the water will run out.

Others, medical men of great experience in aural surgery, say, "*do not pour water* either cold or lukewarn, *habitually* into the ears; the hearing may be impaired by the practice, as the tympanic membrane is most delicate. the wet end of a towel or silk handkerchief is all that is sufficient." For my own part, I prefer immersing the whole head, while washing and letting water run into both ears, while the head is in the basin or else, after washing or sponging the face and eyes, if the basin be not big enough and deep enough to admit the whole head beneath water, then by turning the head first on one side, and then on the other in the basin, to admit the water in the ears, which comes to much the same thing; but it is not quite so refreshing.

Then sponge the chest and small of the back, and underneath the arm pits; this is the least you ought to do. (I advocate sponging the whole body, and that both night *and* morning.) The least you can do with any attention to cleanliness or health, is to sponge the face, chest and back with water, and dry-rub the rest of the body, every morning, if not evening. Eschew the ridiculous practice of standing half naked, soaking and washing your hands for half an hour, the sole notion a boy has of washing himself. If you use a tooth brush night and morning, as you should do, it cannot be too soft. Hard brushes make the gums flee from the teeth, and produces premature decay, by causing the soft bone of the tooth to be exposed to the air, beyond the part of the tooth protected by the enamel.

For a thorough wash of the hands, use warm water; let it be rain, not hard or spring water; and before you begin soaping them, steep them well in the water for a minute or two, rubbing them the while, till the water has penetrated well into the pores; then use soap and a nail brush *ad libitum*. And by holding your hands under a tap, if you have one, of cold water, and "give them a shower bath," so to say; it is very refreshing and strengthening to the fingers. If you have no tap, just dip them into cold water, and rub them dry as quick as you can. Miss Nightingale, in her most useful "Notes upon nursing," reccommends as even more effectual, holding them for a few minutes over a jug of hot water, so as to let the steam thoroughly penetrate the seams and pores of the skin, before commencing to soap them. But this requires time.

